# Phenotypic Variability in Synthetic Biology Applications: Dealing with Noise in Microbial Gene Expression

**DOI:** 10.3389/fmicb.2016.00479

**Published:** 2016-04-08

**Authors:** Lucia Bandiera, Simone Furini, Emanuele Giordano

**Affiliations:** ^1^Laboratory of Cellular and Molecular Engineering “S. Cavalcanti”, Department of Electrical, Electronic and Information Engineering “G. Marconi”, University of BolognaCesena, Italy; ^2^Department of Medical Biotechnologies, University of SienaSiena, Italy; ^3^BioEngLab, Health Science and Technology, Interdepartmental Center for Industrial Research, University of BolognaCesena, Italy; ^4^Advanced Research Center on Electronic Systems, University of BolognaCesena, Italy

**Keywords:** synthetic biology, biological noise, intrinsic/extrinsic stochasticity, network topology, standard biological parts

## Abstract

The stochasticity due to the infrequent collisions among low copy-number molecules within the crowded cellular compartment is a feature of living systems. Single cell variability in gene expression within an isogenic population (i.e., biological noise) is usually described as the sum of two independent components: intrinsic and extrinsic stochasticity. Intrinsic stochasticity arises from the random occurrence of events inherent to the gene expression process (e.g., the burst-like synthesis of mRNA and protein molecules). Extrinsic fluctuations reflect the state of the biological system and its interaction with the intra and extracellular environments (e.g., concentration of available polymerases, ribosomes, metabolites, and micro-environmental conditions). A better understanding of cellular noise would help synthetic biologists design gene circuits with well-defined functional properties. *In silico* modeling has already revealed several aspects of the network topology’s impact on noise properties; this information could drive the selection of biological parts and the design of reliably engineered pathways. Importantly, while optimizing artificial gene circuitry for industrial applications, synthetic biology could also elucidate the natural mechanisms underlying natural phenotypic variability. In this review, we briefly summarize the functional roles of noise in unicellular organisms and address their relevance to synthetic network design. We will also consider how noise might influence the selection of network topologies supporting reliable functions, and how the variability of cellular events might be exploited when designing innovative biotechnology applications.

## Introduction

Novick and Weiner (1957) first observed the differential ability of individual *Escherichia coli* cells within an isogenic population to respond to environmental conditions. Since then, the stochasticity due to the infrequent collisions among low copy number molecules subjected to Brownian motion within the cellular compartment has been identified as an inherent feature of living systems (Shahrezaei and Swain, 2008). Owing to its pivotal role in biological processes, stochasticity in gene expression has been the focus of research fostered by the progress in quantitative single-cell assays. Both experimental and theoretical studies have elucidated the prime causes of phenotypic variability and their impact on microbial fitness (Maloney and Rotman, 1973; Spudich and Koshland, 1976; Berg, 1978).

**FIGURE 1:**
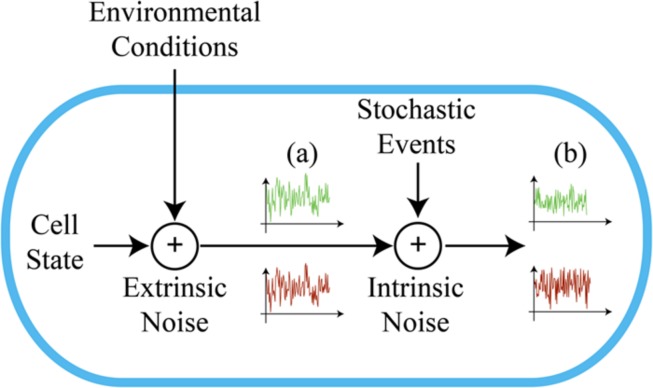
**Intrinsic and extrinsic components of noise.** Extrinsic noise results from changes in the environmental conditions and cellular state. This contribution equally affects two fluorescence reporter proteins transcribed from the same promoter (a). Their expression is modulated in an uncorrelated way by intrinsic noise, due to the stochasticity of biochemical events (b).

The overall variability in gene expression within an isogenic population (i.e., biological noise) is usually described as the sum of two independent components: intrinsic and extrinsic stochasticity (**Figure 1**). Intrinsic stochasticity arises from the random occurrence of biochemical events inherent to the gene expression process (e.g., the burst-like synthesis of mRNA and protein molecules). Extrinsic fluctuations reflect the state of the cell and its interaction with the intra- and extracellular environments (e.g., the concentration of available polymerases, ribosomes, metabolites, and the micro-environmental conditions). These two components have been empirically distinguished either through dual-reporter gene assays (Elowitz et al., 2002; Swain et al., 2002), or via indirect methods (Ozbudak et al., 2002; Blake et al., 2003; Acar et al., 2005). Although extrinsic stochasticity appears to often be the dominant component of biological noise (Elowitz et al., 2002; Raser and O’Shea, 2004; Rosenfeld et al., 2005), we lack a precise characterization of its significant contributors (Shahrezaei et al., 2008; Hilfinger and Paulsson, 2011). On the other hand, the data collected in prokaryotes shows that intrinsic fluctuations relate primarily to translational efficiency (Elowitz et al., 2002; Ozbudak et al., 2002). Synthetic biology would certainly benefit from a quantitative understanding of cellular noise, given that its aim is the design of gene circuits with well-defined functional properties. While optimizing artificial gene circuitry for industrial applications, synthetic biology might also contribute to the understanding of the natural mechanisms underlying phenotypic variability.

In this review, after a brief summary of the functional roles of noise in unicellular organisms, we will discuss its relevance in the design of synthetic networks. In particular, we will consider how noise might influence the selection of network topologies, and how the variability of cellular events might be exploited when designing innovative biotechnology applications.

## Noise in Natural Networks

**FIGURE 2:**
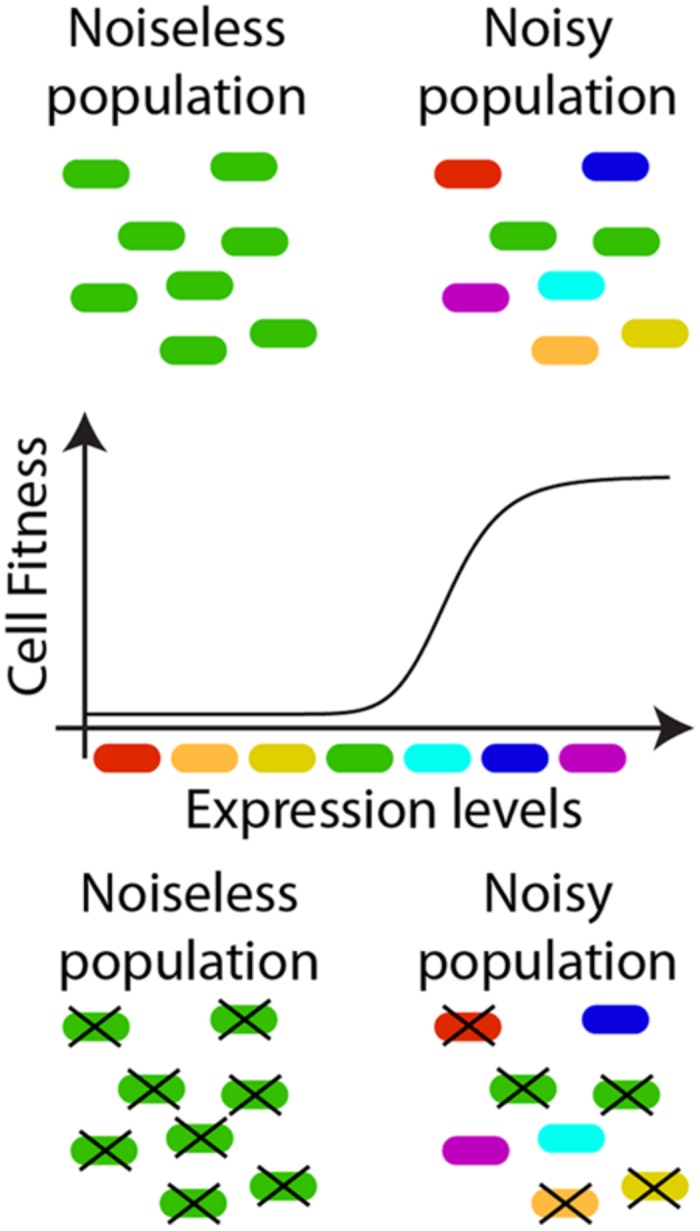
**Noise as a positive regulator of cellular behavior.** Variability in protein expression levels among isogenic individuals might improve the overall performance of a cell population. Boxes of different colors are used to represent cells where a protein (or a set of proteins), critical for the cell survival, is differently expressed among the individuals of an isogenic population. When the population is featured by limited phenotypic variability (left hand side of the lower diagram), different cells have comparable expression levels. In a noisy population (right hand side of the lower diagram), the same average expression level, as accessible with bulk measurements, result from a more dispersed distribution of protein concentrations at the single-cell level. If the cell survival depends on the expression level of a given protein (i.e., only cells with expression levels corresponding to light-blue, blue, and purple boxes survive) a noisier population might be more robust to changes in environmental conditions causing selective stress, thereby ensuring the species survival. Similar strategies could be adopted to improve sensing properties in synthetic applications. 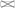 dead; 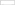 alive. The reader is referred to (Smits et al., 2006) for additional examples.

Noise is generally considered to hamper the outcome of cellular processes relying on fine control of molecular fluxes (Arias and Hayward, 2006). However, a plethora of studies has attributed beneficial functions to noise-driven phenotypic variability (**Figure 2**). For example, the noise in gene expression introduces phenotypic heterogeneity within clonal populations, allowing species survival in time-varying environments. Indeed, fluctuations might divide a clonal population into phenotypic subpopulations, providing an evolutionary advantage without the burden of sensing and reacting (Kussell and Leibler, 2005). A classic example of this logic is represented by the phage λ choice between lytic and lysogenic cycles (Arkin et al., 1998). The phage’s probabilistic fate commitment has been attributed to the overwhelming abundance of one of two key repressors (Cro/CI), interacting through nested positive and negative feedback loops constituting a genetic switch (Dodd et al., 2005; Balazsi et al., 2011). This switch controls a bistable system in which the phenotype decision is memorized in each cell, preventing reversion of fate commitment (Losick and Desplan, 2008).

Another case of noise-driven differentiation is observed in *Bacillus subtilis*; a fraction of the population becomes competent after entering a stationary phase as a stress response induced by limited nutrient availability. This dynamic transition is triggered when the expression level of the regulator *comK* exceeds a threshold value, leading to the activation of downstream genes responsible for the uptake of extracellular DNA. The noisiness of the system impacts both the percentage of cells entering the competent state and its duration (Maamar et al., 2007). Natural variability in the duration of competence events has been related to the architecture of the molecular loop controlling the stress response. Indeed, a rewired network where the end of competence events occurs at high ComS concentrations, rather than at low ones as in the wild-type configuration, exhibits a reduced variability in their duration while preserving the behavior in the deterministic limit. This evidence suggests that cells have evolved mechanisms for tuning and exploiting biological noise within a defined spatial and temporal frame. Note also that noise control is often encoded in simple network topologies, where nested positive and/or negative feedback loops support the coexistence of alternative states and ensure the stochastic achievement of a functional optimum for at least a proportion of the cells, in physiological and pathological conditions (Davidson and Surette, 2008).

## Noise in Synthetic Biology

Synthetic biology seeks to implement *de novo* cellular tasks or rewire faulty cellular processes by engineering complex biological architectures. Many biomolecular widgets are described in the literature (Elowitz and Leibler, 2000; Gardner et al., 2000; Rinaudo et al., 2007; Ham et al., 2008; Win and Smolke, 2008; Friedland et al., 2009; Ceroni et al., 2010, 2012). However, significant effort is still required to achieve a level of complexity (e.g., number of genes composing the network and its organization in hierarchical structures) commensurate with the natural biological landscape in order to offer biosynthetic devices of environmental, industrial and medical relevance. The design of novel gene circuits would greatly benefit from the development of standard procedures for a meticulous and context-dependent characterization of synthetic biological parts and modules. The improved reliability of the parameters describing the properties of and the interactions among these parts would allow the development of more reliable computational models. As a result, the functionality of a device could be accurately predicted and only robust gene circuits would merit physical implementation. Circuits able to operate reliably in noisy conditions (random fluctuations in the environment and in the cell state) require that stochasticity in gene expression be controlled and, ideally, exploited. In addition, the comparison of engineered networks with their natural counterparts would extend our knowledge of design principles shaped by evolution.

Using genetically modified organisms (GMOs), Ozbudak et al. (2002) experimentally investigated the impact of transcription and translation on phenotypic variability. The transcription rate was tuned by means of an inducible promoter or by mutating the promoter sequence, while translational regulation was achieved by inserting point mutations in either the ribosome binding site (RBS) or the first codon of a green fluorescent protein (GFP) reporter. Noise amplitude was reduced when translation of GFP was driven by an inefficient RBS, confirming earlier theoretical predictions that translational efficiency is a major determinant of prokaryotic gene expression noise. In the same year, Elowitz et al. (2002) evidenced that noise magnitude scales with increasing promoter strength and that the relative contribution of intrinsic and extrinsic components to the overall stochasticity varies with the expression regime. Indeed, when the fluorescent reporters’ expression was downregulated intrinsic noise monotonically decreased (Paulsson and Ehrenberg, 2001; Swain et al., 2002), while extrinsic fluctuations reached a maximum at intermediate transcription rates. Thus, strong promoters and weak RBS sequences should be selected when assembling robust gene circuits (Libby et al., 2007; Lehner, 2008). Recently, the tuning of active promoter-specific transcription factors has also been proposed as a useful tool to define the sensitivity and dynamic range of inducible promoters as well as the noise profile of the associated genes. Weak (strong) promoters driving the transcription of the repressor (activator) enhanced both sensitivity and dynamic range of the regulated promoter. Noise amplitude proved coherent with expectations at low induction (e.g., low titers of receptor were associated with high stochasticity) and decreased at higher inducer concentrations (Wang et al., 2015).

Another variable relevant for noise control is the gene circuit copy number, with high copy-number plasmids reducing the relevance of finite number effect. Indeed, increasing plasmid copy number implements the synthetic analog of evolutionary gene redundancy, a strategy adopted in naturally occurring networks, which enhances their robustness (Raj et al., 2006; Kafri et al., 2009). However, one has to consider the metabolic burden imposed on transformants by plasmid high copy number; moreover, the variation in their counts during cell growth and division could introduce an additional source of extrinsic stochasticity (Paulsson and Ehrenberg, 2001), complicating the relationship between the copy number and the amplitude of noise.

Clearly, the architecture of a network also impacts its dynamics and robustness. In fact, regulatory mechanisms that cells evolved to tune noise (Swain, 2004; Pedraza and Paulsson, 2008; Lestas et al., 2010) are implemented through complex networks. Their properties have been theoretically and experimentally investigated, permitting the characterization of elementary synthetic circuits such as feedback loops and transcriptional cascades.

Positive feedback loops, in which a protein upregulates its own synthesis, have been associated with bistability and increased phenotypic variability. The bimodal distribution of protein levels reflects the coexistence of high- and low-expression states, between which single cells stochastically switch. Analyzing the positively regulated expression of a GFP reporter in *Saccharomyces cerevisiae*, Becskei et al. (2001) attributed the lack of an ON/OFF switch to a hysteretic component, which could reduce GFP fluctuations by ‘remembering’ past states. An inverse proportionality between positive feedback strength and switching frequency has been theoretically proved (Isaacs et al., 2003).

The general idea that negative feedback loops enhance system robustness while reducing gene expression noise (Thattai and van Oudenaarden, 2001; Rao et al., 2002; Simpson et al., 2003; Orrell and Bolouri, 2004) has been experimentally demonstrated by Becskei and Serrano (2000), who compared fluctuations in TetR-GFP levels controlled by a PTet promoter. The less noisy behavior was observed at maximal feedback strength, while administering anhydrotetracycline resulted in weaker feedback and noisier expression. In contrast Dublanche et al. (2006) observed optimal noise suppression at intermediate feedback strengths. Their results agreed with theoretical analyses indicating that negative feedback has the ability to reshape the noise spectrum through a shift from low to high frequency components. The latter can easily be suppressed by downstream molecular cascades acting as low-pass filters. In particular the extent of the shift, a function of the feedback strength, was maximum at intermediate strengths (Austin et al., 2006).

The most prevalent form of negative feedback in natural networks is protein-mediated transcriptional regulation (Alon, 2007; Balazsi et al., 2008; Nevozhay et al., 2009; Singh and Hespanha, 2009). Alternative negative-feedback topologies can be implemented through transcriptionally-/translationally regulated expression of a gene mediated by mRNA (Zhang et al., 2005, 2008; Bandiera et al., 2016). In fact, mRNA-operated translational gene downregulation is indicated as the best noise suppression strategy by mathematically controlled comparison of efficiency in alternative regulatory mechanisms of noise minimization. It is worth noting that the disruption of this type of negative feedback has been associated with pathological states and improper stress-related responses (Hinske et al., 2010; Schmiedel et al., 2015). Although mRNA-based feedback proved optimal for minimizing noise under the constraint of fixed feedback strengths, it is important to consider that, when the protein products translated from the target mRNA regulate the strength of the feedback via their multimerization, this introduces a cooperative regulation which might render transcription/translation ultrasensitive to protein levels.

The effect of the length of a transcriptional cascade on noise propagation has been investigated by Hooshangi et al. (2005), who compared the magnitude of fluctuations in networks with up to three stages. The authors observed higher stochasticity at intermediate inducer concentrations, revealed by bimodal fluorescent distributions. Furthermore, the addition of a transcriptional layer approximately doubled gene-expression noise, resulting in the noisiest output at maximal cascade length. The increasing number of stages improved the hypersensitivity of the network at intermediate induction, leading to a more precise steady-state switch between low and high expression levels, but it also extended the time required for network activation. This caused decreased synchronization within the population, as transient intercellular variability in the activation times increased. Analogous results were obtained by Blake et al. (2003) and Pedraza and van Oudenaarden (2005). Remarkably, theoretical studies showed that elongating a transcriptional cascade leads to low-pass filter activity, preventing network activation from short, noisy inputs (Powell, 1958).

## Exploiting Noise in Synthetic Circuits

Thanks to a deeper understanding of the strategies adopted to modulate the amplitude and the spectral properties of biological noise in nature, it is now possible to develop more reliable synthetic devices. Indeed, the usefulness of engineered systems in applicative contexts requires the precise integration of multiple inputs providing details on extracellular environment and host internal state. Lu et al. (2008) developed a theoretical noise generator, in which the independent regulation of transcription and translation of any gene of interest allows its expression at different noise levels while preserving mean protein concentration. Coupling the output of such a generator with a designed network input would allow us to test the robustness of synthetic circuits. Furthermore, understanding the effect of stochasticity on the expression of crucial transcription factors would hasten the development of optimal phenotypic reprogramming strategies. This has been verified in a broad range of settings, including innovative cancer therapies where neoplastic cells are specifically targeted by lytic phages synthesizing chemotherapeutic agents (Lu et al., 2009) and protocols to selectively knock down cancer-related cascades (Xiang et al., 2006).

Natural phenotypic variability in isogenic populations also challenges the use of GMOs in industrial processes, where it reduces their yield (Alonso et al., 2012; Lee et al., 2013). In addition to stochasticity in gene expression, genetic mutations and heterogeneous extracellular environments might also contribute to this unwanted outcome. Genetic mutations, which could overcome the productive population, increase with culture time. A solution for this issue might come from active biocontainment procedures, used to prevent the spread of GMOs in the natural environment. Thus, heterologous genetic circuitry might include cell-cycle dependent promoters which drive the expression of a toxic protein after a given number of replications (Lu et al., 2009), thereby constraining the age of the vital population and preventing the useless nutrients consumption by undesired and potentially unproductive mutants. Heterogeneity in batch and fed-batch bioreactors’ environment (such as gradients in carbon sources, oxygen, carbon dioxide and pH) exposes cells to sudden variations in extracellular signals, introducing an uncontrollable and history-dependent cell-to-cell variability. This latter effect might be counteracted by re-designing bioreactors or developing engineered strains robust to these oscillations (Neubauer and Junne, 2010). Due to the slow diffusion of chemical signal, the environmental gradients characterizing biofilms – in which GMOs have shown to be more robust and efficient than planktonic equivalent (Li et al., 2006; Gross et al., 2007) – support phenotypic variability which might underpin functional stratification. Nevertheless, experimental studies suggesting the benefits of using biofilms compared to stirred reactors have so far been performed at the micro-scale level and might therefore not be preserved with the scale-up.

Biochemical noise also constitutes a practical concern in the engineering of metabolic pathways. Dynamic control of the enzyme’s expression would allow an adjustment of the synthetic pathway state to variations in host metabolism and bioreactor environment, thereby preventing metabolic flux imbalance and insufficient yields. Hypothesizing the metabolite-dependent transcriptional downregulation of an enzyme operating in a catalytic reaction, Oyarzun et al. (2015) numerically examined the effect of synthetic-circuit parameter space on noise propagation in a metabolic pathway. The authors found that weak promoters and high negative feedback strength (or vice versa) together minimize noise levels. Their work emphasized the usefulness of *in silico* predictions for selecting biological parts and circuit topology. It is worth noting that dynamic control in heterologous expression systems has theoretically (Dunlop et al., 2010) and experimentally shown to enhance population-level efficiency through optimization of single cells pathway flux balance (e.g., phenotypic noise). Indeed, *E. coli* toxic intermediate-responsive promoters outcompeted constitutive or inducible analogs of equal strength when comparing the yields in the mevalonate-based isoprenoid biosynthetic pathway (Dahl et al., 2013). In another study, the dynamically controlled expression of the ion-efflux pump *eilA* counteracted the growth-inhibiting effect of ionic solvents used in pre-treatment of carbon biomasses, leading to a more effective biofuel production (Frederix et al., 2014). Considering the theoretical analysis previously reported, we envisage that an mRNA-based downregulation of enzyme synthesis, e.g., via riboregulators, might lead to a faster-responding and less noisy equivalent. The coupling of synthetic circuits’ control with cell–cell communication strategies (e.g., quorum sensing) is perhaps the best known and pursued example of harnessing phenotypic variability within an isogenic individuals in order to achieve integrated and coordinated population behavior. This possibility has been explored in You et al. (You et al., 2004), where a quorum-sensing system was coupled with a negative feedback to control cell-density, via tuning of cell death rate, as determined by the expression level of the *toxic* ccdB gene. In absence of intercellular variability, the synthetic circuit would have failed in allowing the attainment of a stable cell density, as the whole population would have died at high levels of the killer gene.

## Conclusion

The application of synthetic biology tools to investigate different components of stochasticity has provided insight into the regulatory mechanisms that tune biological noise in natural networks. The efficiency of these mechanisms has inspired the selection of biological parts and topologies that could control the behavioral dispersion of engineered micro-organisms. Although further efforts are required, especially for the characterization of extrinsic components, enhancing network robustness could foster the design of innovative synthetic devices, with potential benefits in bioremediation, biomedicine and biotechnology.

## Author Contributions

LB drafted the manuscript. All the authors critically read, edited, and approved the document.

## Conflict of Interest Statement

The authors declare that the research was conducted in the absence of any commercial or financial relationships that could be construed as a potential conflict of interest.
